# Preparation of a Carbon Doped Tissue-Mimicking Material with High Dielectric Properties for Microwave Imaging Application

**DOI:** 10.3390/ma9070559

**Published:** 2016-07-09

**Authors:** Siang-Wen Lan, Min-Hang Weng, Ru-Yuan Yang, Shoou-Jinn Chang, Yaoh-Sien Chung, Tsung-Chih Yu, Chun-Sen Wu

**Affiliations:** 1 Department of Electrical Engineering and Advanced Optoelectronic Technology Center, Institute of Microelectronics, National Cheng Kung University, Tainan 701, Taiwan; s907952271@gmail.com (S.-W.L.); changsj@mail.ncku.edu.tw (S.-J.C.); 2 Medical Devices and Opto-Electronics Equipment Department, Metal Industries Research and Development Center, Kaohsiung City 811, Taiwan; hcwweng@gmail.com (M.-H.W.); tcyu@mail.mirdc.org.tw (T.-C.Y.); csw@mail.mirdc.org.tw (C.-S.W.); 3 Graduate Institute of Materials Engineering, National Pingtung University of Science and Technology, Pingtung County 912, Taiwan; t2891962@gmail.com

**Keywords:** dielectric property, oil-in-gelatin, microwave image, tissue-mimicking material (TMM), carbon

## Abstract

In this paper, the oil-in-gelatin based tissue-mimicking materials (TMMs) doped with carbon based materials including carbon nanotube, graphene ink or lignin were prepared. The volume percent for gelatin based mixtures and oil based mixtures were both around 50%, and the doping amounts were 2 wt %, 4 wt %, and 6 wt %. The effect of doping material and amount on the microwave dielectric properties including dielectric constant and conductivity were investigated over an ultra-wide frequency range from 2 GHz to 20 GHz. The coaxial open-ended reflection technology was used to evaluate the microwave dielectric properties. Six measured values in different locations of each sample were averaged and the standard deviations of all the measured dielectric properties, including dielectric constant and conductivity, were less than one, indicating a good uniformity of the prepared samples. Without doping, the dielectric constant was equal to 23 ± 2 approximately. Results showed with doping of carbon based materials that the dielectric constant and conductivity both increased about 5% to 20%, and the increment was dependent on the doping amount. By proper selection of doping amount of the carbon based materials, the prepared material could map the required dielectric properties of special tissues. The proposed materials were suitable for the phantom used in the microwave medical imaging system.

## 1. Introduction

Microwave imaging (MWI) technology attracts much attention in medical use and seems an alternative imaging modality for the well developed modalities such as X-ray, ultrasound, or magnetic resonance imaging (MRI) due to some advantages including light and portability, low-cost, non-ionizing radiation, and imaging without the need of contrast agents [1,2,3,4,5]. The tissue-mimicking materials (TMMs) with desired microwave dielectric properties including dielectric constant and conductivity are thus developed for the microwave medical imaging techniques in the application of mimicking the human tissue and calibrating the imaging systems. Many different materials have been presented to prepare the TMMs, such as polyacrylamide gel (PAG), oil-in-gelatin, and carbon-based synthetics [6,7,8,9,10,11,12]. PAG is typically comprised of Acrylamide (C_3_H_5_NO) polymerized in liquid solvent and used to simulate the behavior of biological tissues in applicators for microwave hyperthermia or in scaled-phantom experiments, with the advantages including excellent optical transparency and high elasticity [6,7]. However, the fabricated phantoms using PAG showed a short usage life with only several hours as exposed to air or several weeks as held in a tight-covered container. 

Compared with the above materials, gelatin-based materials are popular because of the simple fabrication, stable mechanical properties and a long usage time [8]. The gelatin-based materials mixed with oil were typically used especially to imitate the dielectric properties of the human soft tissues, since they could provide a high competitiveness for the controllable and tunable characteristics of dielectric properties by varying the concentration of oil [9,10]. Moreover, the oil-in-gelatin based TMMs have long-term stability in heterogeneous configurations without change in geometry or dielectric properties. However, the microwave dielectric properties of the oil-in-gelatin based TMMs could not be tuned significantly by varying a small quantity of oil. Therefore, the dielectric properties of the prepared TMMs usually provided a small tuning range through varying the volume percent of oil in general.

In another carbon-based material, using urethane as the matrix material mixed with graphite and carbon black, was newly adopted to fabricate the microwave phantom of mimicking fatty tissues, providing the characteristics of stable and flexible dielectric properties [11,12]. Carbon nanotubes are known as electronic conductive materials and can be used to vary the dielectric properties in composition. They exhibit a high dielectric constant at microwave frequency due to their fibrous shapes [13]. Similarly, graphene is also an electronic conductive material and possesses good electrical and mechanical properties due to the unique structure of two-dimensional aromatic sheets [14]. The lignin, extracted from the renewable resource material, is suggested as an ionic conductive material and typically has high electrical resistance due to the absence of unsaturated bonds. However, the components of lignin are highly hydrophilic and might have high polarization. Lignin has been proposed as a low cost carbon material with good mechanical properties [15,16]. Moreover, the carbon based materials typically have a general characteristic of electromagnetic wave absorption at high frequency spectrums, which leads to a slow wave and influences the dielectric properties [13,14]. Based on the above information, it is desired to know the doping effect of the carbon based materials, such as carbon nanotube, graphene ink, and lignin, on the preparation of oil-in-gelatin based materials.

To develop a new tissue-mimicking material for phantom, many issues shall be known, including the preparation method, uniformity, measurement accuracy, measured dielectric properties, mechanical properties and stability. In this paper, we focused on the doping effect of the carbon-based synthetic materials on the microwave dielectric properties of the oil-in-gelatin based TMMs. To clear the doping effect, the gelatin and oil were both fixed at the volume percent of 50%. The prepared samples were measured over an ultra-wide frequency range from 2 GHz to 20 GHz. It was found that the microwave dielectric properties of the studied samples were tuned significantly by varying small doping amounts of the carbon based materials. The preparation and measurement were presented in detail as following, and the microwave dielectric properties of the prepared samples were investigated and discussed in this paper.

## 2. Methodology

### 2.1. Preparation of the Tissue-Mimicking Materials

**Figure 1:**
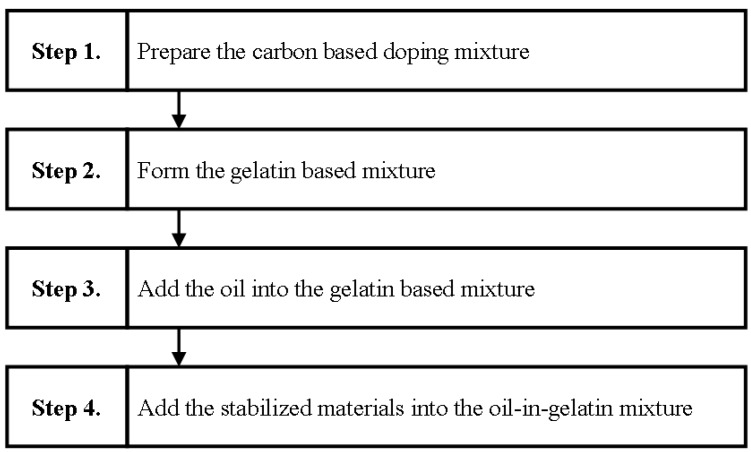
The main procedure of preparing the proposed oil-in-gelatin based materials doped with carbon based materials.

In general, the investigated materials were mainly a mixture of gelatin and oil, thus still regarded as the oil-in-gelatin based TMMs. In this study, the volume percent for gelatin based mixtures and oil based mixtures were both around 50%. To investigate the doping effect on the microwave dielectric properties, three carbon based materials, comprising carbon nanotubes, graphene ink, or lignin, were added individually into the oil-in-gelatin based TMMs during the preparation procedure. The weight percent of the added carbon based materials were decided as the variation factors to tune the dielectric properties of the prepared samples, and chosen as 2, 4, and 6 wt %. For comparison purposes, we prepared a sample without doping carbon nanotubes, graphene ink, or lignin, which was expressed as 0 wt % in the plotted diagram. The mixed oil was prepared by combining 50% kerosene and 50% safflower oil. To illustrate the preparing procedure of the proposed samples simply, the main preparing steps are summarized as shown in Figure 1. It is noted that the four main steps are generalized based on the fabrication procedure of oil-in-gelatin based TMMs [9]. However, after doing many experiments to find the method of doping carbon based materials into the oil-in-gelatin, it was found that the doping materials should be added into the mixed solution under vigorous stirring before adding gelatin in order to spread the carbon based materials uniformly in the prepared samples, as shown in Step 1. Thus, additional steps are adopted because of the doping of carbon based materials.

**Figure 2:**
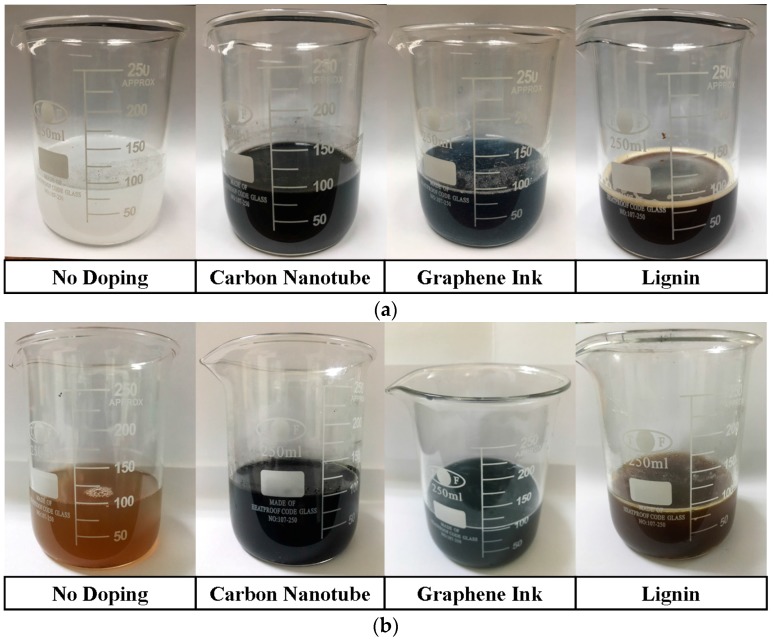

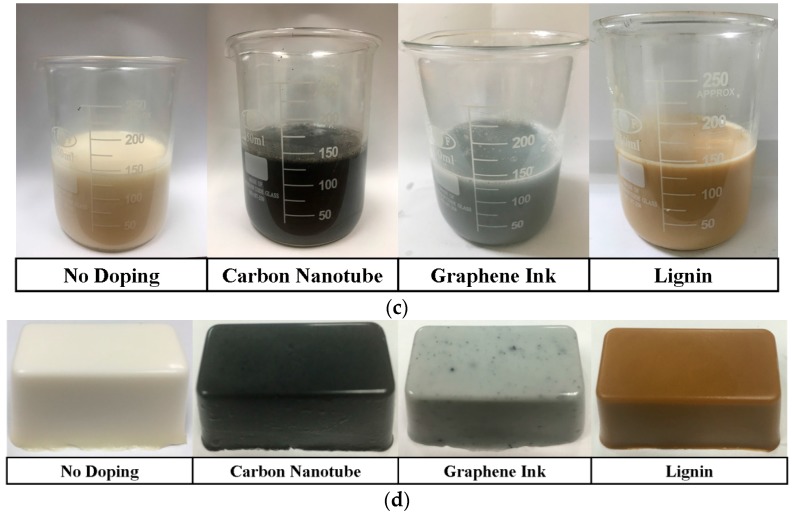
The variation of typical prepared and studied samples (**a**) mixed solutions; (**b**) water-gelatin mixtures; (**c**) oil-gelatin mixtures and (**d**) the solidified samples. The doping concentrations of carbon based materials are schematically chosen as 6 wt %.

The detailed procedure of preparing the proposed samples, as extended from the main four steps in Figure 1, is illustrated as below [9]. Herein, the variation of typical prepared and studied samples is shown in Figure 2.

- During step 1:

Prepare 0.8 g of n-propanol in a beaker, mix the n-propanol with 0.2 g of p-toluic acid (powder) and heat the solution until the p-toluic is completely dissolved.

Mix the solution with 19 g of deionized (DI) water (18 Mohms∙cm of resistivity).

Add the carbon based materials, including carbon nanotube (95% concentration, CDW-181, Taiwan Carbon Nano Technology Corporation, Miaoli County, Taiwan), graphene ink (0.5% concentration, G1208, Legend Star International Co. Ltd., New Taipei City, Taiwan), or lignin (371017, Sigma-Aldrich, Shanghai, China), individually into the solution, and stir the solution by using a magnetic stirrer at about 600 rpm at room temperature for 10 minutes (mixed solutions are shown in Figure 2a).

- During step 2:

Add 3.5 g of gelatin (G9391, Sigma-Aldrich, Shanghai, China), which was extracted from bovine skin Type B, into the solution to obtain a water-gelatin mixture. 

Cover the beaker, containing water-gelatin mixture, with a plastic film, and heat the beaker by double-boiling at about a temperature of 90 °C.

Remove the beaker from the double-boiling system after the gelatin is completely dissolved, and take off the plastic film. In this stage, hold the temperature of the beaker at 50 °C by a hot plate to avoid the water-gelatin mixture solidifying (water-gelatin mixtures are shown in Figure 2b).

- During step 3:

Prepare 15.5 g of mixed oil (50% kerosene and 50% safflower oil) in another beaker and preheat it to 50 °C. 

Pour the mixed oil into the beaker, containing water-gelatin mixture, to obtain an oil-gelatin mixture (oil-gelatin mixtures are shown in Figure 2c).

- During the step 4:

Add 8.6 g of liquid surfactant (Ultra Ivory, Procter & Gamble Professional, Cincinnati, OH, USA) and 0.08 g of formaldehyde into the oil-gelatin mixture, and stir mixture violently by using magnetic stirrer at about 800 rpm at temperature of 50 °C.

Pour the oil-gelatin mixture into a container for solidifying and shaping after the oil-gelatin mixture becomes uniform and there are no bubbles remaining beneath the surface. It is noted that the container was in a sealed condition to avoid particles attaching on the surface of the oil-gelatin mixture for at least five days at room temperature (the solidified samples are shown in Figure 2d).

In this study, to increase usage lifetime of the prepared samples, the formaldehyde is used to preserve the sample, and the weight of the formaldehyde is chosen as 0.22 g per gram of gelatin. The surfactant is used to reduce the interfacial tension between the oil and water, and the weight of the surfactant is chosen as 0.55 g per gram of oil. For different doping materials, the weight percentages of all component materials of the prepared TMMs are list on Table 1.

### 2.2. Measurement of the Tissue-Mimicking Material

**Figure 3:**
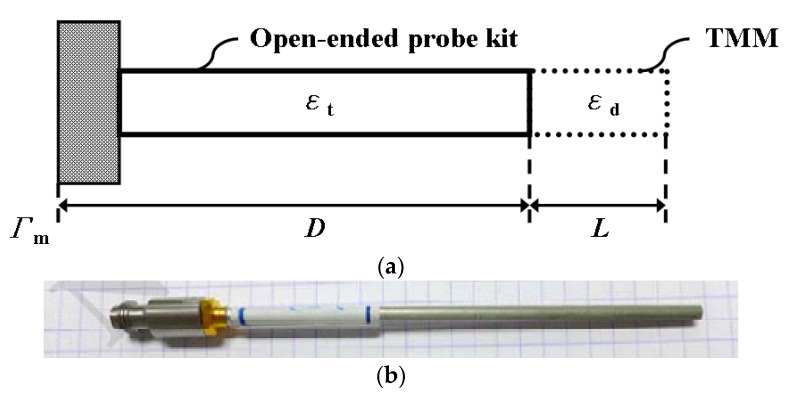
(**a**) The equivalent structure and (**b**) practical structure of the coaxial open-ended reflection probe.

The microwave dielectric properties of the prepared samples were measured and determined using the coaxial open-ended reflection technology. Figure 3 shows (a) the equivalent structure and (b) practical structure of the coaxial open-ended reflection probe. The dielectric properties of the samples can be expressed as Equation (1) [17]:

(1) $$\varepsilon_{d}=\frac{-jc\sqrt{\varepsilon_{t}}}{2\pi fL}\frac{1-\Gamma_{m}e^{2\gamma_{t}D}}{1+\Gamma_{m}e^{2\gamma_{t}D}}(\frac{j2\pi fL\sqrt{\varepsilon_{d}}}{c})\coth(\frac{j2\pi fL\sqrt{\varepsilon_{d}}}{c}),$$

and the unknown length (*L*) can be expressed as Equation (2) [18]:

(2) $$L=\frac{c}{j2\pi f\sqrt{\varepsilon_{water}}}\tanh^{-1}(\frac{1-\Gamma_{water}e^{2\gamma_{t}D}}{1+\Gamma_{water}e^{2\gamma_{t}D}}\frac{\sqrt{\varepsilon_{t}}}{\sqrt{\varepsilon_{water}}}),$$

where some constants are known first including: *c* is the light speed in vacuum (*c* is 3 × 10^8^ m/s), *ε*_t_ is the dielectric constant of the probe kit (*ε*_t_ = 2.03), *D* is the length of the probe kit (*D* = 112 mm), *ε*_water_ is the dielectric constant of water (*ε*_water_ = 78.3), and some symbols are the measured results including: *f* is the operated frequency, *Γ*_m_ is the measured reflection coefficient of the probe kit, *Γ*_water_ is the reflection coefficient of water, and *γ*_t_ is the propagation constant. To extract the real value of the unknown length (*L*), an iterative calculation was done by substituting the known dielectric constant of air and water [18].

**Figure 4:**
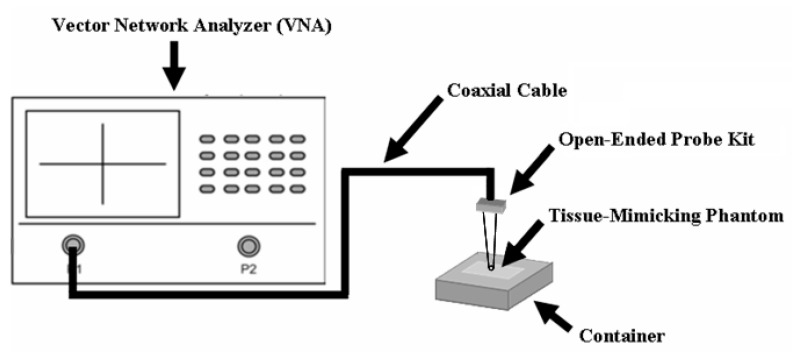
Schematic structure of the experimental setup for the measurement of dielectric properties.

The measurement system for microwave dielectric properties of the TMMs in this study were set by using an Agilent 8722ES Vector Network Analyzer (VNA) (Santa Clara, CA, USA) combined with the open-ended probe kit, as shown in Figure 4. One side of the open-ended probe kit was connected to the port of VNA through a coaxial cable, and the other side touches the surface of the prepared TMMs to take the S-parameter. Before connecting the open-ended probe kit to the end of the coaxial cable, the coaxial cable was connected to three calibration kits (open kit, short kit, and match kit) individually for the calibration. Three calibrated results were extracted and calculated by VNA to ensure the accuracy during the measuring period. Herein, electronic calibration kits 85056D (Agilent, Santa Clara, CA, USA) were used to calibrate the coaxial cable. The expected accuracy of the dielectric probe measurement technique is with maximum differences of 10% or less between measured dielectric properties and values derived from published theoretical Debye models, as discussed in [18].

**Figure 5:**
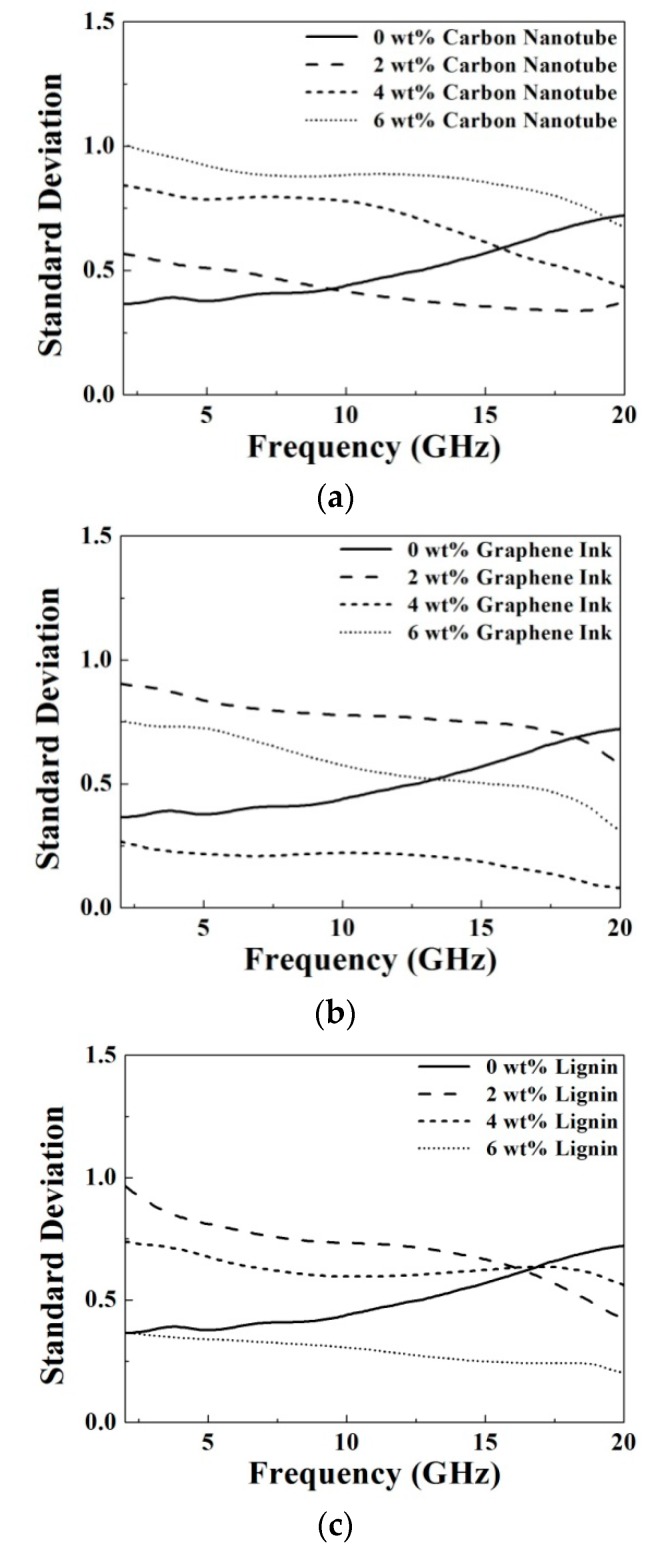
The calculated standard deviation of the measured dielectric constant for the prepared samples with different doping materials, including (**a**) carbon nanotube; (**b**) graphene ink and (**c**) lignin.

**Figure 6:**
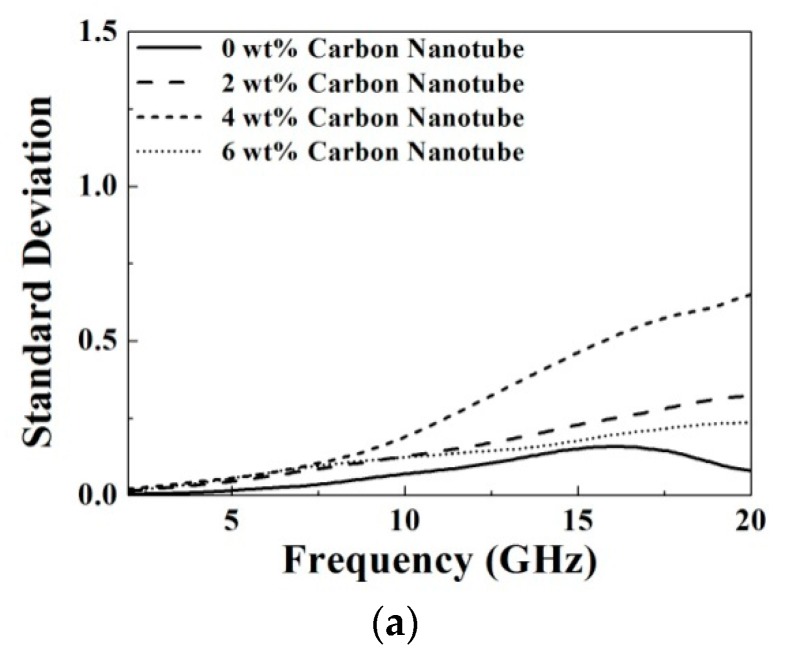

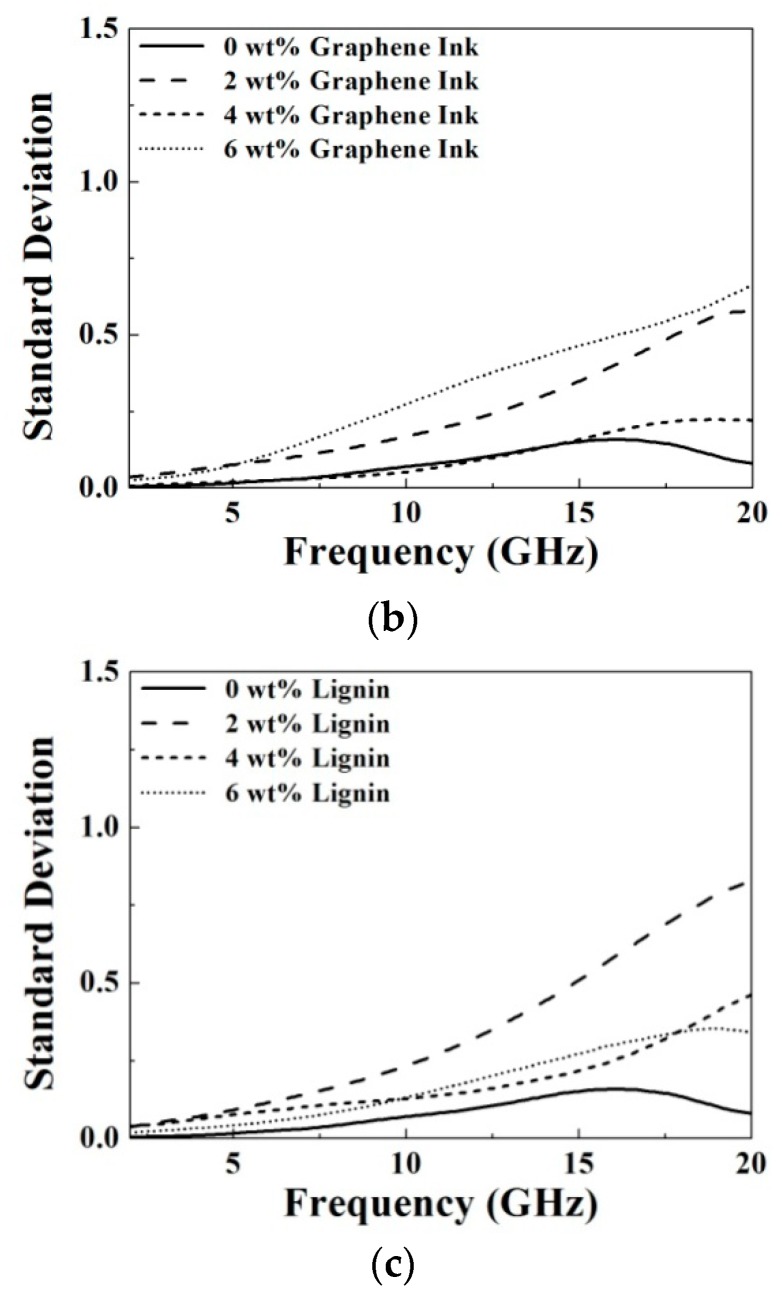
The calculated standard deviation of the measured conductivity for the prepared samples with different doping materials, including (**a**) carbon nanotube; (**b**) graphene ink and (**c**) lignin.

It shall be noted that the probe end was pressed to contact the surface of the prepared samples slightly without puncturing it when measuring [18]. To enhance the accuracy of the measured results, during the measuring period, each sample was measured at six different positions under the same environment condition at room temperature of 26 °C, and then all the six measured values were averaged and recorded. Moreover, the standard deviations (SDs) of the dielectric properties were calculated to evaluate the uniformity for each sample. The formula of SD for each sample can be expressed as:

(3) $$SD=\sqrt{\frac{1}{6}\sum_{i=1}^{6}{\left(x_{i}-\bar{x}\right)}^{2}},$$

where *SD* is the value of standard deviation, *x*_i_ is the measured result of the dielectric properties, and $\bar{x}$ is the averaged result of six recording dielectric properties. Figure 5 and Figure 6 show the SD of dielectric constant and conductivity versus the frequency for the prepared samples with different doping materials. Obviously, the calculated values of SD of all the measured dielectric properties, including dielectric constant and conductivity, are less than 1, indicating a good uniformity of the prepared samples.

## 3. Results and Discussion

**Figure 7:**
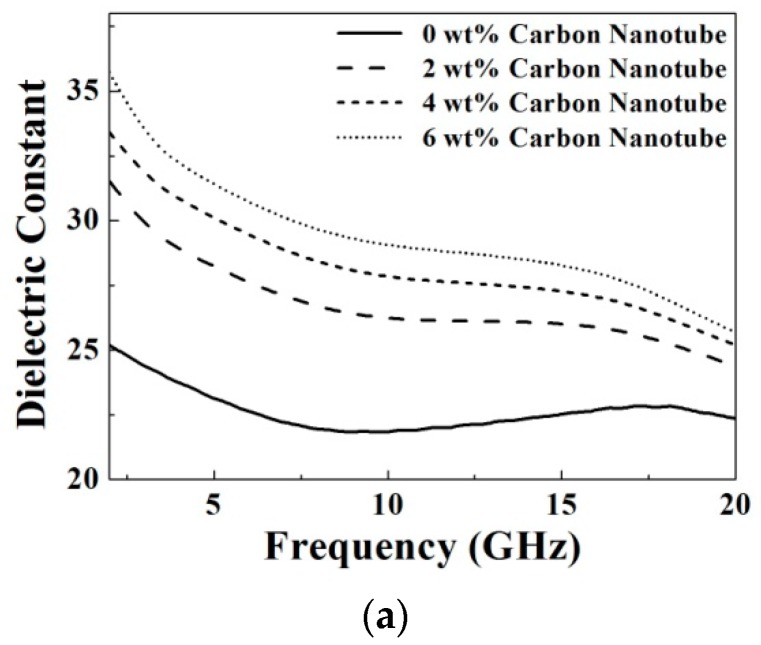

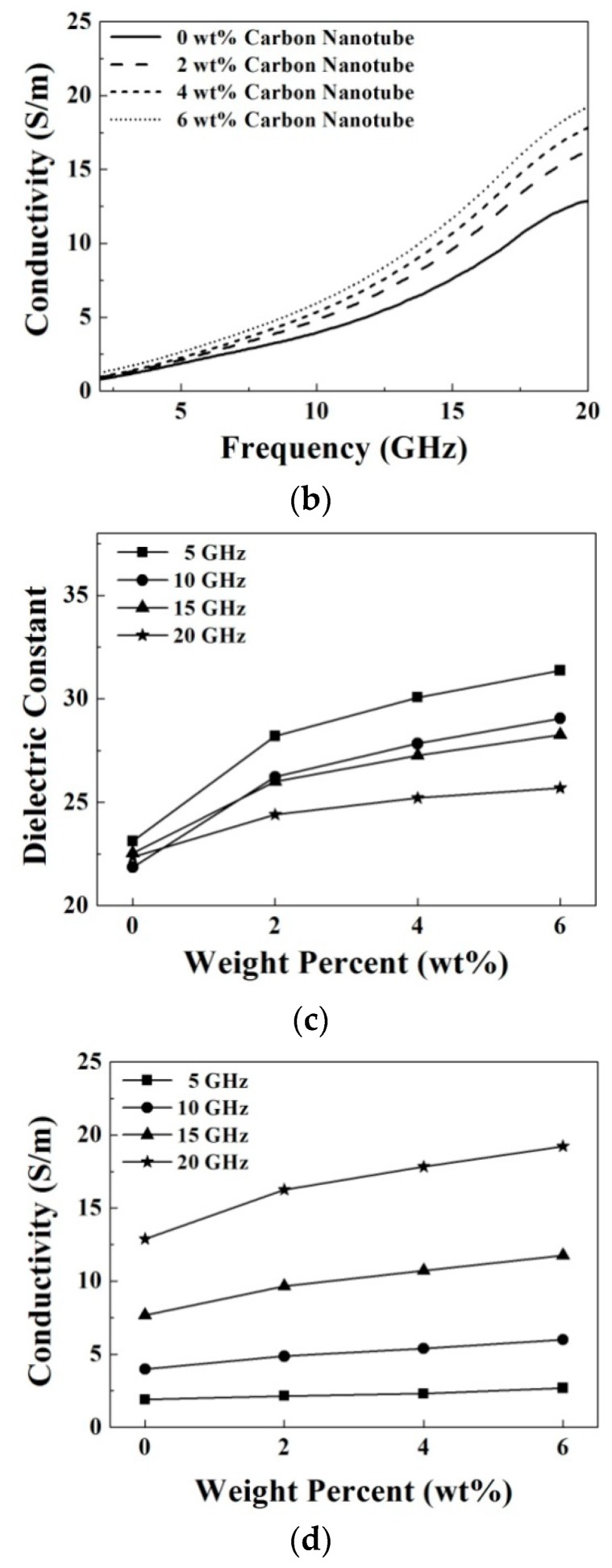
(**a**) Dielectric constant; (**b**) conductivity; (**c**) increment variation of the dielectric constant, and (**d**) increment variation of the conductivity of the prepared samples as functions of the doping concentrations of carbon nanotube.

Figure 7 shows the measured microwave dielectric properties at a frequency range from 2 GHz to 20 GHz as functions of the doping concentrations of carbon nanotube. It is observed that with increasing the carbon nanotube concentration from 0 wt % to 6 wt %, the dielectric constant and conductivity both increase at a fixed frequency as shown in Figure 7a,b. This result might be contributed by the parasitic capacitor effect, formed by gaps between the carbon nanotubes due to the operating frequency at microwave spectrum, as discussed in [19]. As doping of 0 wt %, 2 wt %, 4 wt % and 6 wt %, the dielectric constants are 21.8, 26.2, 27.8 and 29.1, respectively, and the conductivities (S/m) are 3.9, 4.9, 5.4 and 6.0, respectively, at 10 GHz. The increment variation of the dielectric constant and conductivity are summarized in Figure 7c,d. It is clearly found that the increments of the dielectric constant become small but the increments of the conductivity become large while increasing the frequency from 5 GHz to 20 GHz. 

**Figure 8:**
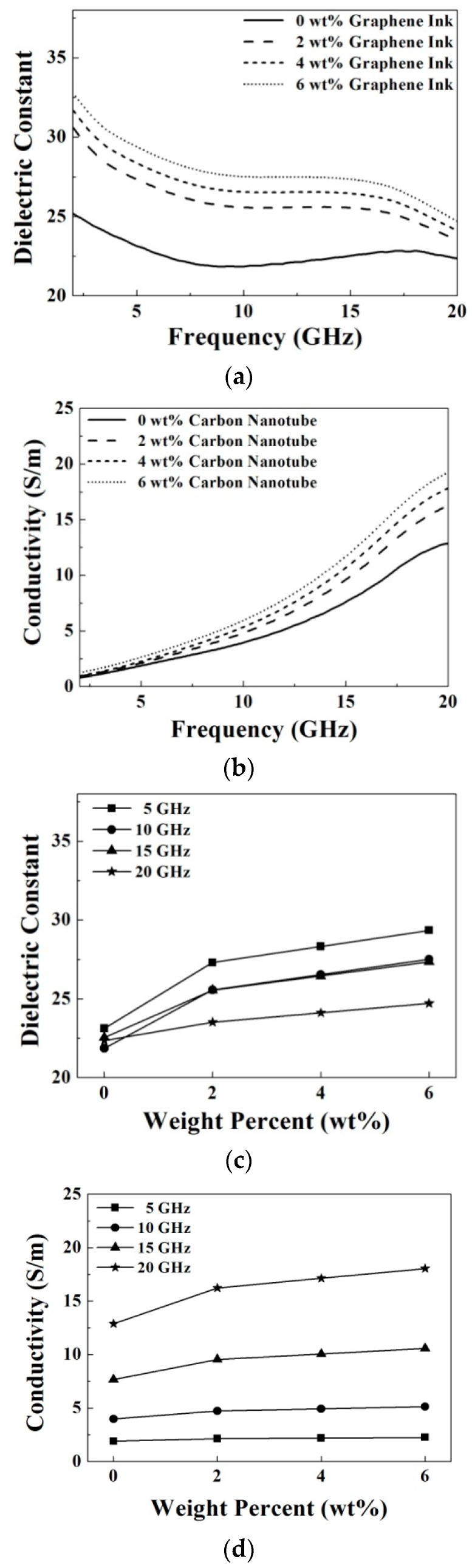
(**a**) Dielectric constant; (**b**) conductivity; (**c**) increment variation of the dielectric constant, and (**d**) increment variation of the conductivity of the prepared samples as functions of the doping concentrations of graphene ink.

Figure 8 shows the measured microwave dielectric properties at a frequency range from 2 GHz to 20 GHz as functions of the doping concentrations of graphene ink. Similarly, it is found that with increasing of the graphene ink concentration from 0 wt % to 6 wt %, the dielectric constant and conductivity both increase at a fixed frequency as shown in Figure 8a,b. This result might also be contributed to by the parasitic capacitor effect, as doping with the carbon nanotubes [19]. As doping of 0 wt %, 2 wt %, 4 wt % and 6 wt %, the dielectric constants are 21.8, 25.6, 26.5 and 27.5, respectively, and the conductivities (S/m) are 3.9, 4.7, 4.9 and 5.1, respectively, at 10 GHz. The increment variation of the dielectric constant and conductivity are summarized in Figure 8c,d. It is shown that the increments of the dielectric constant are small, but the increments of the conductivities are large when increasing the frequency from 5 GHz to 20 GHz.

**Figure 9:**
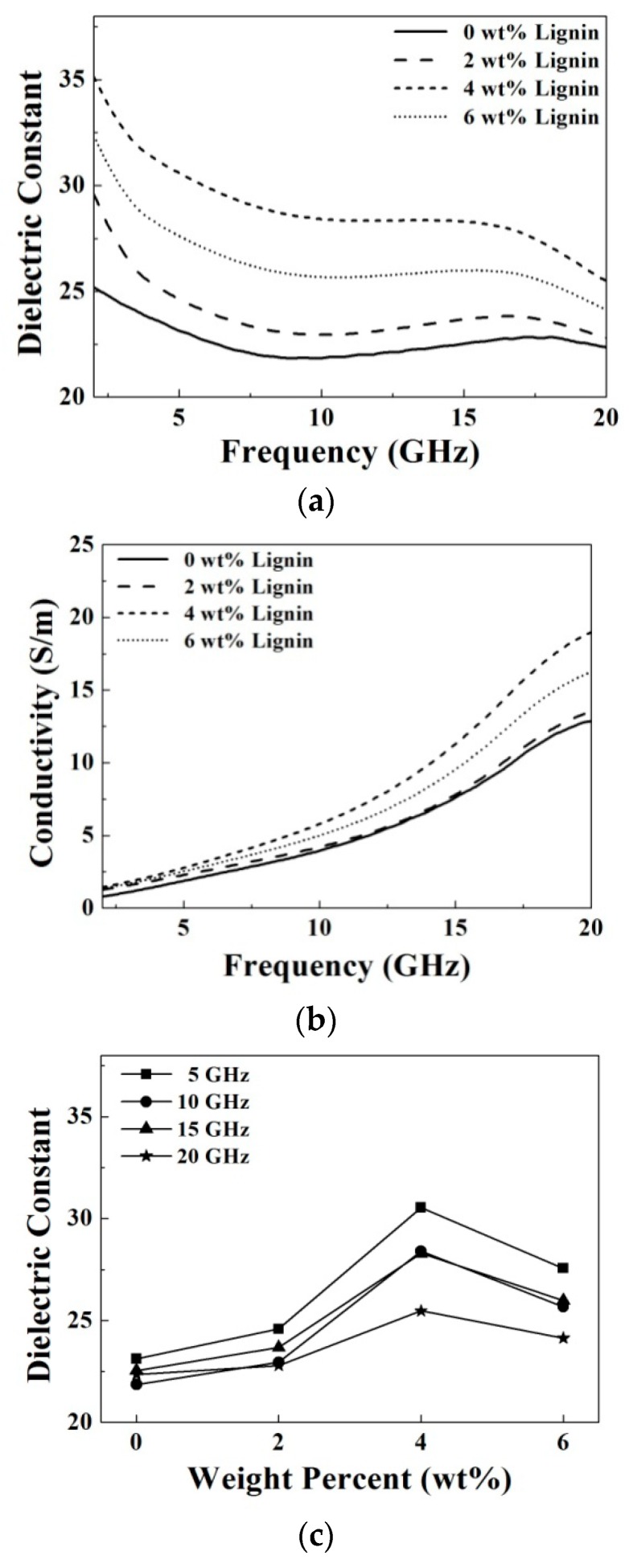

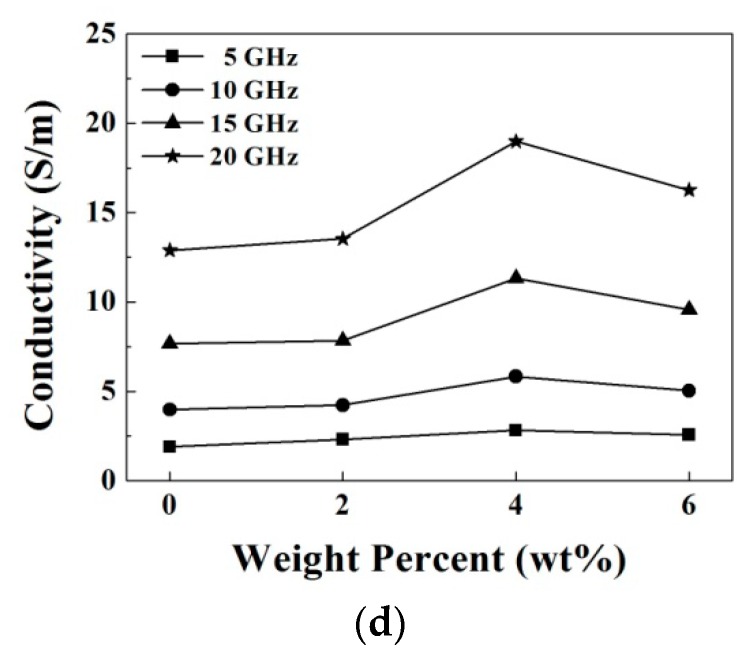
(**a**) Dielectric constant; (**b**) conductivity; (**c**) increment variation of the dielectric constant, and (**d**) increment variation of the conductivity of the prepared samples as functions of the doping concentrations of lignin.

Figure 9 shows the measured microwave dielectric properties at a frequency range from 2 GHz to 20 GHz as functions of the doping concentrations of lignin. It is shown that with increase of the lignin concentration from 0 wt % to 4 wt %, the dielectric constant and conductivity both increase at a fixed frequency, as shown in Figure 9a,b. As doping of 0 wt %, 2 wt %, 4 wt % and 6 wt %, the dielectric constants are 21.8, 22.9, 28.4 and 25.7, respectively, and the conductivities (S/m) are 3.9, 4.2, 5.8 and 5.0, respectively, at 10 GHz. Although the lignin is not an electronic conductive material, the lignin with polysaccharide components is highly hydrophilic and might have high polarization to increase the dielectric properties. The suggested mechanism still needs further study. The increment variation of the dielectric constant and conductivity are summarized in Figure 9c,d. Similar to the doping with carbon nanotube and graphene ink, the increments of the dielectric constant become small, but the increments of the conductivities become large with increase of the frequency from 5 GHz to 20 GHz. However, the doping effect of lignin reaches a maximum increment at 4 wt % in this study. The result is not clear now and might be due to the saturation of the doping effect since too much lignin dispersed in the oil in gelatin based matrices reduces the polarization ability.

Dielectric constant and conductivity of the prepared oil-in-gelatin based materials doped with carbon based materials at the microwave frequency are a function of the frequency, doping material and doping amount. For the samples without doping, the dielectric constant is equal to 23 ± 2 and the conductivity is from 2 to 12 (S/m) over the measured frequency. In order to clear the doping effect on the dielectric constant and the conductivity, it is desired to plot the increment variations, as shown in Figure 7, Figure 8 and Figure 9. 

It is clearly observed that the increment of dielectric constant is larger at a lower frequency and higher doping amount. In general, the dielectric constant increases from about 5% to 20% after increasing the doping amount from 0 to 6 wt %. Comparison between these added carbon based materials, the dielectric constant of the prepared samples are varied significantly by doping a little bit of lignin, but slightly by doping the same amount of graphene ink. For example, at 10 GHz, the dielectric constant increases about 26.6% (from 21.8 to 27.6) after doping 4 wt % carbon nanotube, and the dielectric constant increases about 20.2% (from 21.8 to 26.2) after doping 6 wt % graphene ink. However, the dielectric constant increases about 30.3% (from 21.8 to 28.4) after merely doping 4 wt % lignin.

It is also observed that the increment of conductivity is larger at higher frequency and higher doping amount. However, the increment ratio of conductivity is lower than that of the dielectric constant. Doping of the lignin still has more obvious effect on the dielectric constant and conductivity than the other two carbon based materials. The reason is now not clear and needs further investigation.

**Figure 10:**
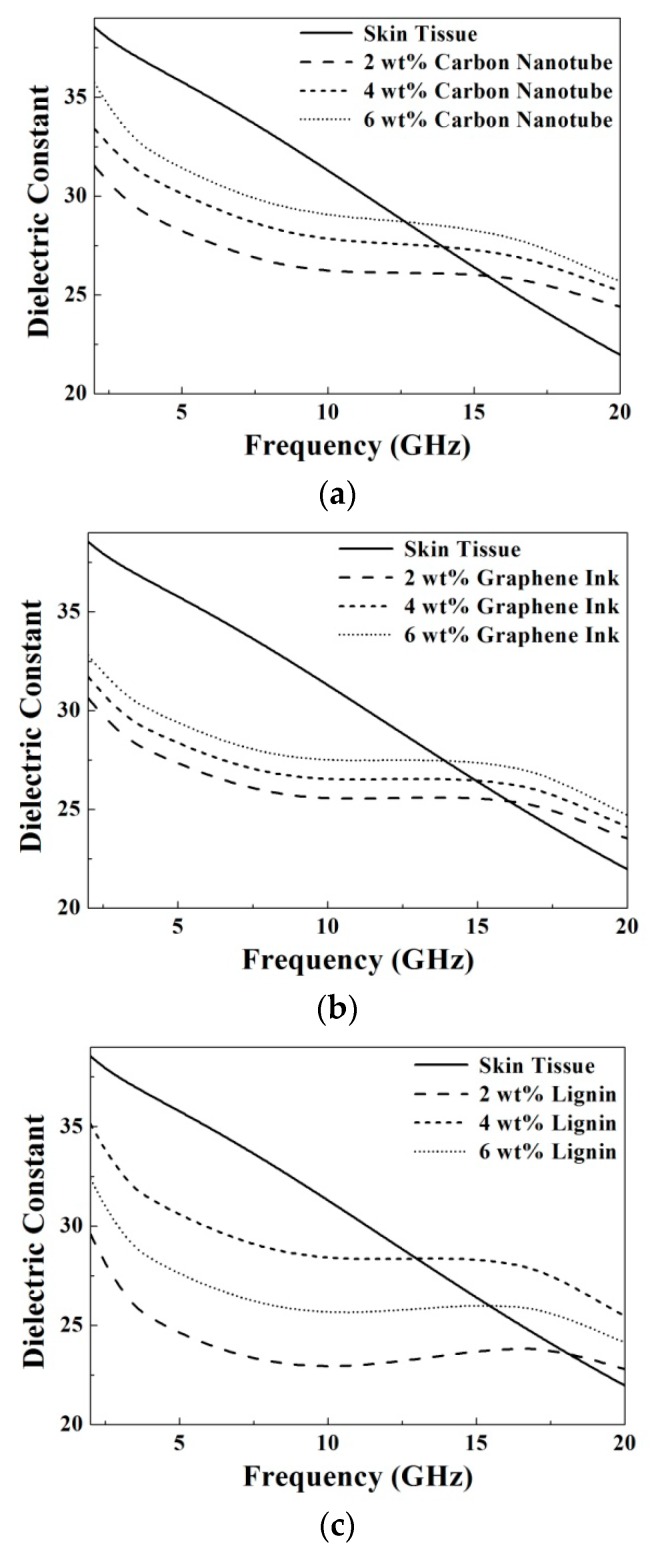
Comparison of dielectric constants between the skin tissue and the prepared samples with different doping concentrations of (**a**) carbon nanotube; (**b**) graphene ink and (**c**) lignin.

**Figure 11:**
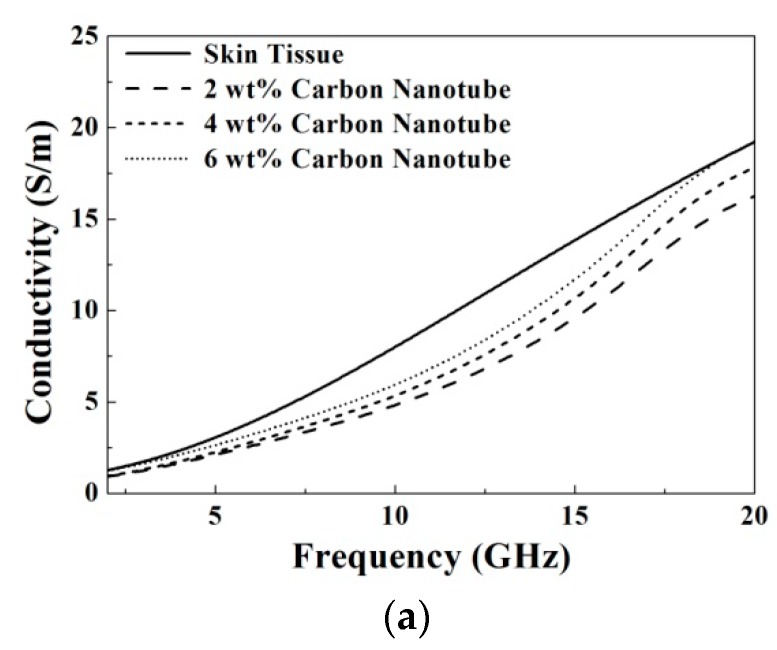

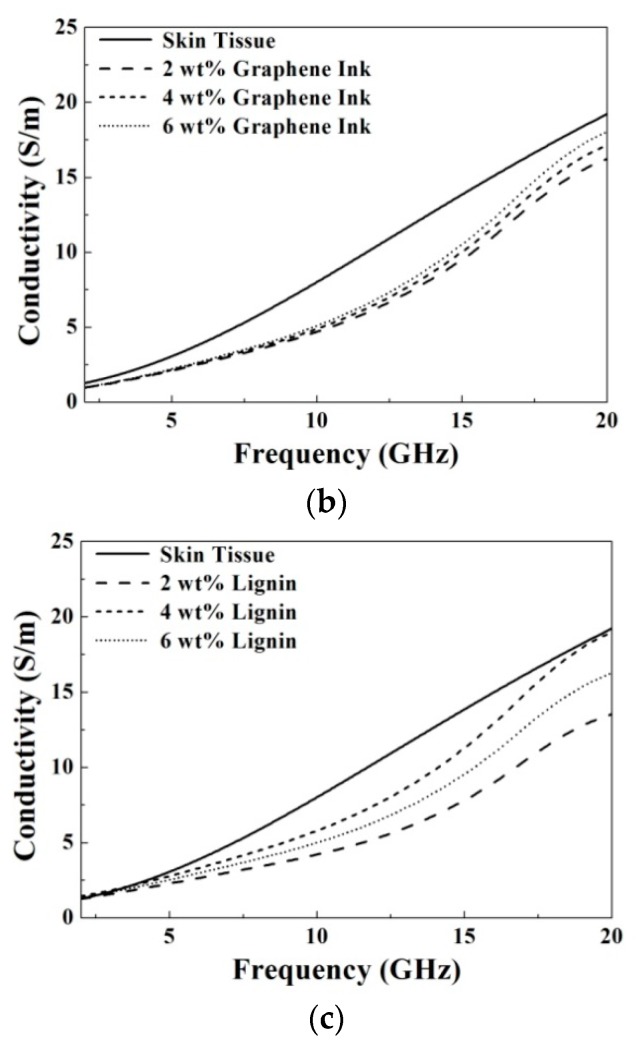
Comparison of conductivities between the skin tissue and the prepared samples with different doping concentrations of (**a**) carbon nanotube; (**b**) graphene ink and (**c**) lignin.

Figure 10 and Figure 11 show the comparison for the microwave dielectric properties of the prepared TMMs and skin tissue. To validate the proposed materials, we compared the dielectric properties of the prepared samples and the biological tissues measured using a four-pole Cole–Cole model summarized and parameterized by Gabriel et al. [20]:

(4) $$\varepsilon (\omega )=\varepsilon_{\infty }+\sum_{n=1}^{4}\frac{\Delta \varepsilon_{n}}{1+{\left(j\omega \tau_{n}\right)}^{1-\alpha_{n}}}+\frac{\sigma_{i}}{j\omega \varepsilon_{0}},$$

where *ε*_∞_ is dielectric constant in the infinite frequency, *ω* is the angular frequency, *τ* is relaxation time, and *σ* is the ionic conductivity. By mapping the required microwave dielectric properties of the skin tissue, the doping quantities of carbon nanotube, graphene ink, and lignin were chosen optimally as 6 wt %, 6 wt %, and 4 wt %, respectively. The results exhibit that all of the prepared TMMs, doped by carbon nanotube, graphene ink, and lignin, are suitable to mimic the skin tissue by controlling the microwave dielectric properties effectively.

In our sample, the dielectric properties are stable and unchanged within two weeks after preparation. It was reported that the oil-in-gelatin materials obtained long-term stability [9]. Since the basic matrix of the tissue-mimicking material is oil-in-gelatin, it is expected that the proposed material can have a good stability. We are now still studying comprehensively the effect of the carbon based materials on the mechanical properties and long time stability with different environment conditions such as temperature and humidity.

## 4. Conclusions

In this paper, we have successfully synthesized oil-in-gelatin based materials doped with different amounts of carbon based materials, including carbon nanotube, graphene ink and lignin for the tissue-mimicking use. The dielectric constant and conductivity of the prepared materials both increased by adding more amounts of carbon nanotube and graphene ink due to the parasitic capacitor effect. However, with doping lignin, the dielectric constant and the conductivity both first increased and then slightly decreased, indicating a saturation of the doping effect. The results showed that the dielectric properties of the oil-in-gelatin based material can be further tuned by adding a small amount of the carbon based material and mapping it to the dielectric properties of the special tissue.

## Figures and Tables

**Table 1:** Weight percentages of the each component material of the presented tissue-mimicking phantom. (Unit: wt %).

Doping Materials	N-Propanol	P-Toluic Acid	Di Water	Gelatin	Mixed Oil	Surfactant	Formaldehyde
2	1.651	0.041	39.2	7.221	31.979	17.743	0.165
4	1.617	0.04	38.4	7.074	31.326	17.381	0.162
6	1.583	0.04	37.6	6.926	30.674	17.019	0.158
